# Management of an Extremely Low Birth Weight Infant with Bilateral Renal Obstruction Caused by *Candida albicans* Fungus Balls

**DOI:** 10.1155/2019/3684734

**Published:** 2019-11-05

**Authors:** Fabian Brüning, Axel Hegele, Günter Klaus, Rolf F. Maier, Rainer Hofmann

**Affiliations:** ^1^Department of Urology and Pediatric Urology, University Hospital, Philipps-University Marburg, Baldingerstr. 1, 35043 Marburg, Germany; ^2^KfH Pediatric Kidney Center and Department of Pediatrics, University Hospital, Philipps-University Marburg, Baldingerstr. 1, 35043 Marburg, Germany; ^3^Children's Hospital, Philipps-University Marburg, Baldingerstr. 1, 35043 Marburg, Germany

## Abstract

We report an extremely low birth weight infant with anuria caused by bilateral *Candida albicans* fungus balls it was treated with a combination of antifungal therapy, irrigation and pyelotomy. This lead to a recovery of renal function, after a follow-up of 77 month no more Candida was cultured from urine.

## 1. Introduction

Candida infections in very/extremely low birth weight infants are frequent (up to 16.7% among very low-birth-weight infants (birth weight <1,500 g) and up to 20% among extremely low-birth-weight infants (birth weight <1,000 g)) [1]; renal obstruction caused by fungus balls are rare [2].

Treatment varies from conservative strategies with single or combined antifungal therapies, to drainage with percutaneous nephrostomy with or without local irrigation or open/endoscopic surgical removal of fungal balls [3].

Here a case is reported on the use of a combination of antifungal therapy, urokinase irrigation and pyelotomy of an extremely low birth weight infant with bilateral ureteral obstruction caused by bilateral Candida fungus balls following *Candida septicaemia*.

## 2. Case Report

The male infant was born at an external hospital by ceasarean section at 25 weeks of gestation with a birth weight of 770 g. A broad spectrum of antibacterial and antifungal drugs was administered for recurrent bacterial and fungal infections including septicaemia and meningitis leading to repeated respiratory insufficiencies with the need for artificial ventilation.

On day 58, he again suffered from sepsis and *Candida albicans* was cultured from urine (susceptible for Fluconazole) and blood specimen. Systemic intravenous antifungal treatment with Fluconazole (6 mg/kg every day without a loading dose) was initiated. Nevertheless acute renal failure developed with anuria (serum creatinine 2.02 mg/dl, blood urea nitrogen 18 mg/dl).

For further treatment, the infant was transferred to our neonatal intensive care unit with the diagnosis of acute renal failure secondary to Candida urinary tract infection.

**Figure 1:**
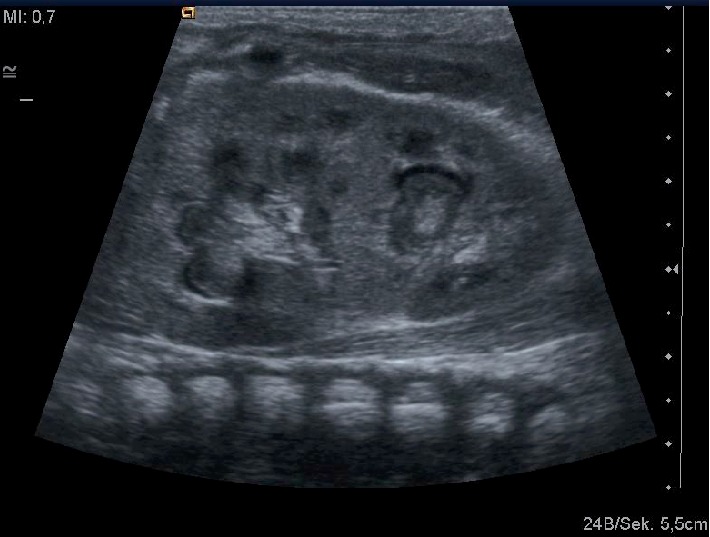
Left kidney with hydronephrosis and fungus balls in the collecting system.

Renal and bladder ultrasonography revealed bilateral dilatation of the collecting systems with echogenic contents within the renal pelvis bilaterally. This was more pronounced on the right side where the content filled the whole renal pelvis including the proximal ureter. Renal parenchyma revealed hyperechogenic, the bladder was empty (Figure 1).

The infant received mechanical ventilation and inotropic support. After stabilization of ventilation and circulation renal replacement therapy by peritoneal dialysis was started via a Tenckhoff catheter.

We changed antifungal treatment to Caspofungin because of a persistence of Candida in urine culture with resistance to Fluconacole. Because of the obstructive effect of the fungus balls an open bilateral pyelotomy was performed. On the right side complete removal of the fungus balls was achieved, whereas on the left side residuals in the upper calices remained due to complex anatomical conditions. On both sides we placed 6 F percutaneous nephrostomy tubes. As early as during this surgery diuresis started again.

Blood urea nitrogen and creatinine levels returned to normal ranges after 7 days with normal diuresis after initial polyuria and peritoneal dialysis was stopped.

From the removed fungal balls *Candida albicans* and Enterobacter cloacae was proven. Enterobacter cloacae was also detected in blood cultures so that antibiotic therapy with vancomycin and meropenem was started.

Antegrade pyelograpy was performed on day 13 after surgery; it showed residuals of fungus balls in the left renal pelvis as a central filling defect and a normal filling of the collecting system on the right side. The right ureter did not show up; on the left side it occurred wormed with passage of contrast media into the bladder.

Because of persisting Candida in the urine culture of the left nephrostomy we started local irrigation with Amphotericin-B solution (50 mcg/ml in saline) for 14 days in addition to the intravenous therapy with caspofungin.

In addition, we started on both sides with local irrigation of urokinase solution (15,000 IU/ml 2x/d) to dissolve local adherence due to inflammatory tissue responses on the right side and to dissolve the residues of fungus balls on the left side.

**Figure 2:**
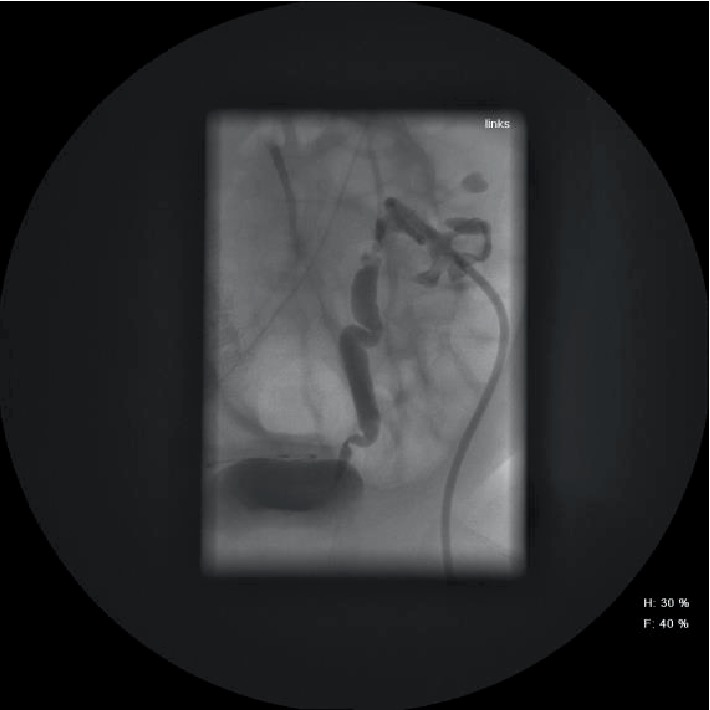
Antegrade pyelography, left ureter wormed and segmentally dilated, residues of fungus balls in the collecting system.

A next antegrade pyelography was performed 7 days after starting the local irrigation therapy and showed a passage of contrast media on both sides into the bladder. The right ureter was thin, the left ureter looked still wormed and segmentally dilated. On the left side residues of fungus balls were still detectable (Figure 2).

After collecting sterile urine cultures after 14 days of irrigation therapy we removed both nephrostomy catheters and the Tenckhoff-catheter. The systemic caspofungin therapy was stopped 28 days after it has been started. No serious side effects associated with this treatment were observed.

Repeated urine cultures continued to yield *Candida albicans*. Therefore oral Fluconazol therapy was continued until month 56. Thereafter no *Candida albicans* were cultured from urine even after stop of the antifungal treatment. At the last follow up at 77 month of life, renal function was stable with serum creatinine of 0.39 mg/dl, leading to an normal estimated creatinine clearance (134 ml/min∗1.73 m^2^).

## 3. Discussion

Candida colonisation has been reported in up to 34% of all infants treated on neonatal intensive care units followed by septicaemia in 7.7% of the cases [4]; urinary tract involvement has been reported in up to 70% of all infants with Candida sepsis [5].

With decreasing birth weight the rate of septicaemia caused by Candida species increases.

*Candida* species are the 3^rd^ most common organism isolated in late-onset sepsis in very low birth weight infants. It is the most common cause of urinary tract infection in neonatal intensive care units [6].

Renal involvement in infants with Candida sepsis presents as parenchymal infiltration and/or fungus balls in the collecting system.

Although urinary candidiasis is a common finding in very low birth weight infants, renal obstruction caused by fungus balls is very rare [2].

Diagnosis of dilatation and fungus balls is done by ultrasound. It shows hyperechogenic material in the collecting system without posterior shadowing [7].

Diagnosis of obstruction can be difficult [8]. In our case anuria and rapidly progressive renal failure suggested complete obstruction so that a combination of systemic and local antifungal drugs, pyelotomy and urokinase irrigation was used to treat an extremely low birth weight infant. Pyelotomy was justified by anatomical circumstances due to the extremely low birth weight.

In the literature, treatment varies from conservative strategies with single or combined antifungal therapies to drainage with percutaneous nephrostomy with or without local irrigation or open/endoscopic surgical removal of fungal balls.

Treatment depends on the presence of complete or incomplete obstruction of the pelviureteric junction [3, 8].

In case of partial obstruction conservative treatment with systemic antifungal medication may be sufficient [8]. Fluconazole is the drug of first choice [9], but treatment should be guided by drug susceptibility of the fungi in urine culture. There is no consensus on the appropriate duration of treatment [10].

In presence of upper urinary tract obstruction systemic antifungal therapy may not be sufficient. Therefore, drainage procedures are indicated with percutaneous nephrostomy being the procedure of choice [8]. This can be a difficult procedure because of the small renal pelvis and the small size of the neonate. In such situations surgical removal of fungus balls with open procedures may be required [11, 12], like in our case.

Sufficient drainage may be enough to clear fungal balls, but mostly systemic antifungal medication is needed additionally. If this is not successful, local irrigation therapy through nephrostomy with fluconazole or amphotericine B was described in some cases to be effective. When local irrigation fails, a fibrinolytic agent (streptokinase or urokinase) has been used successfully in few cases to clear obstructing fungus balls [8].

There is currently no consensus avaible for the treatment of extremely low birth weight infants with obstructive fungus balls.

Clinicians should know the various treatment options, a helpful algorithm for management of these rare cases is given by Bisht and van der Voort [8]. However, treatment still remains an individual decision in each case.

## 4. Conclusion

Candida infections in infants and extremely low birth weight infants are frequent; renal obstruction caused by fungus balls is rare.

In our case the combination of systemic antifungal therapy, urokinase irrigation and pyelotomy lead to a complete recovery of renal function and to a partial removal of the fungal balls with small residuals.

After a follow-up of 77 month no more *Candida albicans* was cultured from urine.

The renal function was preserved and was normal with an estimated creatinin clearance of 134 ml/min∗1.73 m^2^.
